# Polypharmacy in the Older Adult: Medication Review in the Community

**DOI:** 10.1093/geroni/igaa057.674

**Published:** 2020-12-16

**Authors:** Staci Pacetti, Margaret Avallone, Elyse Perweiler

**Affiliations:** 1 Rutgers University-Camden, Camden, New Jersey, United States; 2 Rowan University School of Osteopathic Medicine, Stratford, New Jersey, United States

## Abstract

Polypharmacy remains a significant healthcare issue in the United States, resulting in drug interactions, adverse drug reactions, and potentially dangerous complications. Polypharmacy, often defined as the simultaneous use of five or more medications, may lead to adherence problems and an increased risk of hospitalizations and death, particularly in the older population. Medication management was incorporated into undergraduate nursing clinical experience in an affordable housing urban community as part of the New Jersey Geriatric Workforce Enhancement Program (NJGWEP), a 5-year grant supported by DHHS-HRSA. This paper will describe the first phase of this project, which involved the determination of the prevalence of polypharmacy and high-risk medications in this setting. The charts of sixty residents were reviewed and along with demographic information, the following data was collected: total number of medications (prescription, non-prescription and herbal supplements), high-risk medications using Beers criteria and identification of common themes or issues. The average age of the residents was 72 years, with cardiovascular diseases and diabetes mellitus being the most common comorbidities. Fifty-two of the sixty residents (86%) received five or more medications daily, meeting the definition of polypharmacy. The average number of medications taken on a daily basis was 8.9. Among the residents, centrally-active agents such as gabapentin, tramadol and lorazepam were the most commonly prescribed medications. The second phase of this project will include implementation of a medication reconciliation process to identify potential issues using the Beers criteria and implement appropriate interventions to ensure safe medication practices in this high-risk population.

